# A Comparative Immunohistochemical Expression of TRAP in Odontogenic Cysts

**DOI:** 10.22038/IJORL.2023.63350.3169

**Published:** 2023-11

**Authors:** Hamideh Kadeh, Shirin Saravani, Ali Jamshidi

**Affiliations:** 1 *Oral and Dental Disease Research Center, Department of Oral and Maxillofacial Pathology, School of Dentistry, Zahedan University of Medical Sciences, Zahedan, Iran.*; 2 *Dentist, School of Dentistry, Zahedan University of Medical Sciences, Zahedan, Iran.*

**Keywords:** Dentigerous cyst, Odontogenic Keratocyst, Radicular cyst, TRAP

## Abstract

**Introduction:**

Tartrate-resistant acid phosphatase (TRAP) is an acid phosphatase metalloprotein enzyme expressed in osteoclasts and is related to bone resorption. The molecular mechanisms involved in the different behavior of odontogenic keratocysts have not yet been fully elucidated. The purpose of this study was to compare TRAP expression in odontogenic keratocysts, radicular cysts, and dentigerous cysts.

**Materials and Methods:**

In this cross-sectional study, we selected 60 samples, including 20 cases of each one of the odontogenic keratocysts (OKC), radicular cysts (RC) and dentigerous cysts (DC). The samples were stained with TRAP monoclonal antibodies using immunohistochemistry. The data were analyzed using the Chi-Square and Kruskal-Wallis tests.

**Results:**

In this study, TRAP expression was observed in the lining epithelium of 50% of OKC cases and 5% of RC cases, while it was negative in the lining epithelium of DC. This difference was statistically significant (p<0.001). Moreover, the TRAP staining intensity in the lining epithelium had a significant difference between the groups (P<0.001). TRAP expression in the connective tissue of OKC, RC, and DC was positive in 35%, 30%, and 20% of the cases, respectively. This difference was not statistically significant (P=0.788). Also, staining intensity of TRAP-positive cells in the connective tissue of the lesions was not significant (P=0.634).

**Conclusion:**

In this study, we found a higher expression of TRAP in the lining epithelium of OKC, which may be one of the reasons for the aggressive behavior of OKC compared to other cysts. This finding supports the classification of OKC as an odontogenic tumor.

## Introduction

Odontogenic cysts are among the most common maxillofacial lesions (1), accounting for 7-12% of biopsies in this area according to different studies (2,3). These lesions are classified into developmental types including odontogenic keratocyst (OKC), dentigerous cyst and inflammatory types such as radicular cyst (4). OKC is one of the common jaw cysts with odontogenic epithelium origin, which has aggressive behavior and relatively high rate of recurrence (5,6). It was classified as keratocystic odontogenic tumor (KOT) in WHO 2005 classification; however according to the last edition of WHO Classification of Head and Neck Tumors in 2017, it was reclassified into cystic lesions as there was not sufficient evidence to support the neoplastic nature of this lesion (6-8). Therefore, there have been decades of controversy on classification of OKC as a cyst or a tumor (9). Radicular cyst is the most frequent inflammatory jaw cyst which is formed from the epithelial cell rests of Malassez in periodontal ligament as a result of inflammation (3). It tends to be less than 1.5 centimeters and does not recur if treated properly (10). The dentigerous cyst is the most common developmental odontogenic cyst, accounting for 20% of jaw cysts, and has a low rate of recurrence (11). 

It is believed that cyst formation is related to proliferation of epithelial cell rests of Malassez which are activated by cytokines and growth factors. Immunopathological activities that lead to epithelial proliferation, also induce secretion of bone resorption factors. However, it has not yet been determined that how these processes are involved in the bone formation and resorption. In this regard, expression of factors related to bone metabolism could increase osteolytic activity and extent the cyst into the adjacent bone tissue (12). TRAP (tartrate-resistant acid phosphatase) is a different form of acid phosphatase enzyme with functions in skeleton and the immune system. It is expressed by mono-histocytic cell line including macrophages and dendritic cells. Osteoclasts show staining to TRAP in normal bone and TRAP has been used as an osteoclast histochemistry indicator for years. TRAP is secreted by osteoclasts during bone resorption and formation and is related to resorptive activities (13). Further studies are needed to elucidate the mechanisms involved in the development and aggressive behavior of OKC (14). Currently, researchers are attempting to determine the epithelial and mesenchymal factors, growth mechanism, and the destructive potential of odontogenic lesions such as OKC (11). Several studies have examined the expression of factors related to bone resorption such as RANK/RANKL/OPG in jaw cysts including OKC, RC, and DC (10,12). However, to the authors' knowledge, there are limited studies on TRAP expression (bone resorption indicator) in the odontogenic cysts. Therefore, this study aimed to compare TRAP expression in OKC, RC, and DC.

## Materials and Methods

The present research was approved by the ethics committee of Zahedan University of Medical Sciences (Project No. 2034) (IR.ZAUMS.REC.1396.017). 

This retrospective study was performed using 60 paraffin-embedded tissue blocks of odontogenic cysts consisting of 20 Odontogenic Keratocyst, 20 Dentigerous cyst and 20 Radicular cyst from the Department of Oral and Maxillofacial Pathology, School of Dentistry, Zahedan University of Medical Sciences. At first, H& E slides of the samples were reviewed to confirm the histopathological diagnosis. Information such as age, sex and location of the lesion were obtained from the medical records of the patients. Specimens without sufficient data were excluded from the study. Samples with adequate tissue were selected for immunohistochemical staining.

*Immunohistochemistry*

At first, tissues were prepared in 4µm thickness; deparaffinized with xylene and rehydrated with alcohols solution. Endogenous peroxidase activity was inhibited by 30% hydrogen peroxide-methanol for 30 min. For antigen retrieval, the slides were immersed in citrate solution with PH₌6 for 20 min. The slides were than incubated with primary antibodies of TRAP Clone 26E5 (Novocastra, United Kingdom) according to manufacturer’s instruction. Immune complexes were treated with streptavidin peroxidase (Novolink Polymer Detection System). Immunoreactivity was visualized with diaminobenzidine and was counterstained with Mayer hematoxylin and after drying, the sections were mounted. In negative controls primary antibody was omitted. The TRAP-immunostained cells were evaluated with light microscope (Nikon, Type2, Tokyo, Japan) at a magnification of 100 and 400 in the lining epithelium and connective tissue separately, and expressed as negative or positive (Brown cytoplasmic staining was considered positive). Intensity of staining was scored as: negative (none staining), mild (light brown staining of the cells), severe (dark brown staining of the cells) and moderate (between mild and severe staining of the cells).

*Statistical analysis*

Data was analyzed in SPSS version 21 (SPSS Inc,Chicago, IL) using Chi-square and Kruskal-Wallis test. *P-value* less than 0.05 was considered statistically significant.

## Results

This study examined TRAP expression in 60 cases of odontogenic keratocyst (OKC), radicular cyst (RC), and dentigerous cyst (DC). Demographic information for the study samples is detailed in (Table 1).

**Table 1:** Demographic data of different odontogenic cysts

**odontogenic cysts**	**Age** **mean±SD (year)**	**sex**		**location**	
		**Male** **N (%)**	**Female** **N (%)**	**Maxilla** **N (%)**	**Mandible** **N (%)**
OKC	34.85±14.55	13 (65)	7 (35)	8 (40)	12 (60)
RC	29.8±10.08	12 (60)	8 (40)	12 (60)	8 (40)
DC	23.65±18.02	12 (60)	8 (40)	4 (20)	16 (80)

The immunoreactivity of TRAP was studied separately in the lining epithelium and connective tissue of odontogenic cysts. As shown in table 2, TRAP expression in the lining epithelium was seen in half of the OKC cases (50%) and in one RC case (5%); however, the expression was negative in DC epithelium. Therefore, TRAP expression was significantly higher in OKC compared with other cysts in the lining epithelium (P<0.001). 

**Table 2:** Immunoexpression of TRAP in different odontogenic cysts

**odontogenic cysts**	**epithelium**		**p-value**	**Connective tissue**		**p-value**
	**Positive** **N (%)**	**Negative** **N (%)**		**Positive** **N (%)**	**Negative** **N (%)**	
OKC	10 (50)	10 (50)	<0.001*	7 (35)	13 (65)	0.788
RC	1 (5)	19 (95)		6 (30)	14 (70)	
DC	0 (0)	20 (100)		5 (25)	15 (75)	

*Significant,Chi-Square test

**Fig 1:**
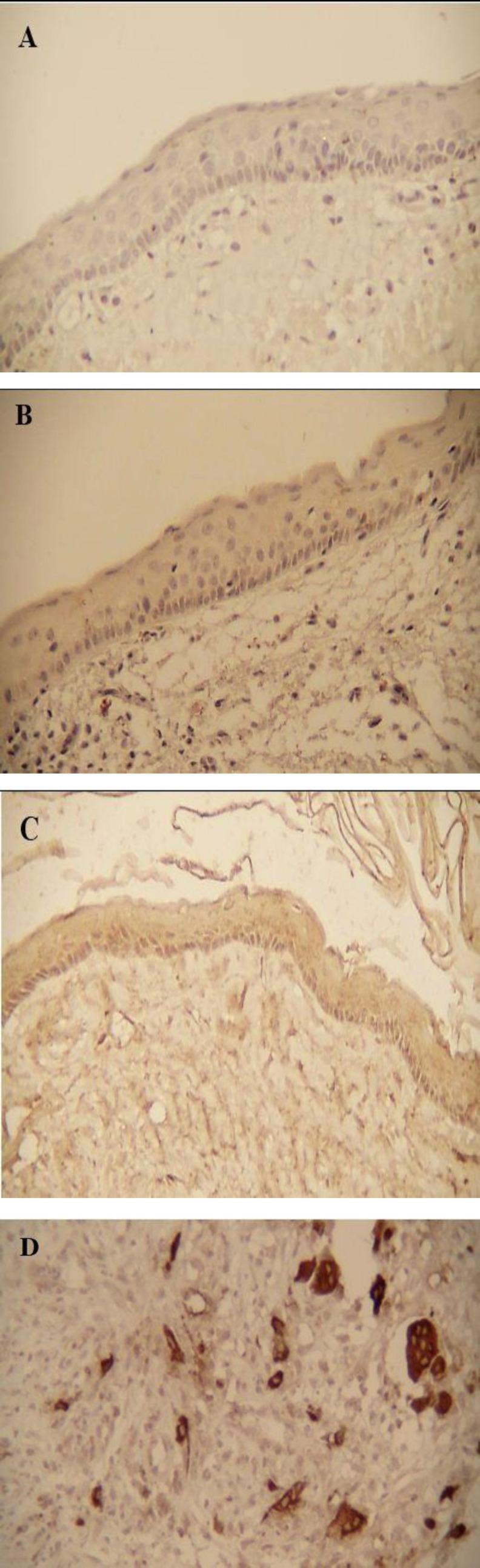
A) Negative expression, B) Mild expression, C) Moderate expression of TRAP marker in the lining epithelium of OKC (×400). D) Positive TRAP expression in the connective tissue of OKC (×400).

**Fig 2:**
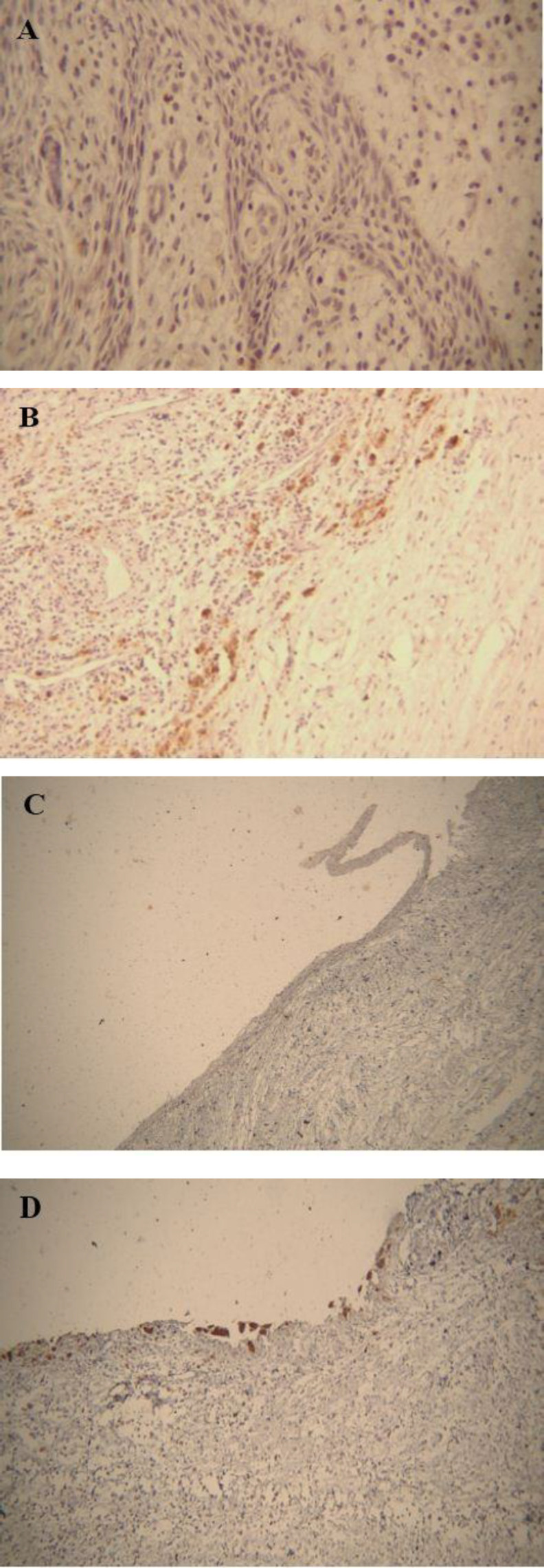
A) Negative expression of TRAP in the lining epithelium of Radicular cyst (×400). B) Positive TRAP expression in connective tissue of radicular cyst (×100). C) Negative expression of TRAP in the lining epithelium of Dentigerous cyst (×100). D) Positive TRAP expression in connective tissue of Dentigerous cyst (×100).

Also, staining intensity of TRAP-positive cells in the lining epithelium of OKC was mild and moderate. This difference between the three groups was significant (P<0.001) (Table3) (Figure 1) (Figure 2). 

**Table 3:** Staining intensity of TRAP in different odontogenic cysts

**odontogenic cysts**	**epithelium**				**p-value**	**Connective tissue**				**p-value**
	**Negative**	**mild**	**moderate**	**severe**		**Negative**	**mild**	**moderate**	**severe**	
OKC	10(50)	7(35)	3(15)	0(0)	<0.001*	13(65)	0(0)	3(15)	4(20)	0.634
RC	19(95)	1(5)	0(0)	0(0)		14(70)	0(0)	4(20)	2(10)	
DC	20(100)	0(0)	0(0)	0(0)		15(75)	1(5)	3(15)	1(5)	

*Significant, Kruskal-Wallis test

TRAP expression in OKC, RC, and DC connective tissue was positive in 35%, 30%, and 20% of the cases, respectively (Table 2). The TRAP expression was higher in OKC connective tissue compared with other cysts, but there was no significant difference (P=0.788). Also, staining intensity of TRAP-positive cells in the connective tissue of the lesions was not significant (P=0.634) (Table 3) (Figure 1) (Figure 2).

In TRAP-positive cases, stained cells were observed throughout the epithelium in OKC. Positive multinucleate and mononuclear TRAP cells were observed in the space between capsule and trabecular bone in the connective tissue of the cysts. Moreover, a large number of positive mononuclear TRAP cells were seen in the perivascular region of the connective tissue of cysts. 

## Discussion

Cysts are formed by the proliferation of epithelial cell rests of Malassez which are activated by cytokines and growth factors. Immunopathological activities that lead to epithelium proliferation simultaneously induce secretion of bone resorption factors(12). Moreover, bone resorption is one of the specific features related to odontogenic cysts (15). 

During bone resorption, osteoclasts secrete enzymes and acids into the space between the osteoclasts border and bone surface. TRAP has been detected both in osteoclasts border and secretions of bone resorption space (16). TRAP staining has been extensively used to detect osteoclast phenotypes in different studies and has been recognized as a reliable marker(15, 16). This study examined TRAP expression in OKC, RC, and DCs. According to the results, TRAP expression in the lining epithelium of OKC was positive in 50% of cases, while it was negative in DCs and there was only one positive case in RCs. Nonetheless, TRAP expression in the connective tissue was seen in all cysts and it was insignificantly higher in OKC compared with RCs and DCs. 

Roh et al.(16) studied TRAP expression and vitronectin receptor, both of which are osteoclast-related cytokines, in ghost cell odontogenic carcinoma. They observed that TRAP expression was present in osteoclasts at the tumor margin. Furthermore, TRAP was detected in ghost cell, but not in tumoral cells. Thus, it was suggested that cytokines secreted from ghost cell such as TRAP and vitronectin receptor, play an important rolein the bone resorption caused by the odontogenic tumor. 

Zecchi-Orlandini et al.(15) studied osteoclast processes of RCs using vitronectin receptor and TRAP. Osteoclast precursors were found in the connective tissue (capsule) which is consistent with our study. It was suggested that similar to bone matrix components spread from resorption surfaces, factors released by radicular cyst could stimulate chemotactic responses from pre-osteoclasts and as a result, help alveolar bone resorption. 

In Formigli et al.(17) study which examined the osteolytic processes involved in radicular cysts, TRAP-positive multinucleated and mononuclear cells were observed at the tip of the intraosseous extensions of the cyst capsule and in direct contact with the bone tissue. The presence of osteoclasts in the space between trabecular bone and cyst capsule suggested that these cells played a significant role in the growth of the lesions within the jaws. Radicular cysts showed TRAP-positive mononuclear cells within the perivascular space of the connective tissue in the cyst capsule. In fact, positive TRAP is not only considered a feature of osteoclasts but also is a cytochemical marker for their mononuclear precursors. These TRAP-positive perivascular cells are probably the osteoclast precursor recruited from circulation within the cyst. It is widely accepted that osteoclast can be formed by the binding of mononuclear precursors with hematopoietic origin and can be used on the bone surface. Therefore, the radicular cyst wall, in addition to releasing osteolytic factors, may also release chemotactic factors to attract pre-osteoclasts into the lesion. 

Tay et al.(18) studied RANKL and TRAP expression in ameloblastoma, DC, OKC, and RC to explain the osteolytic processes in odontogenic cysts and tumors that are mediated by the RANKL pathway. The study showed that the activity of osteoclasts in the connective tissue of lesions significantly contributes to bone destruction. It has been suggested that inflammatory cytokines produced by degenerative bone lesions can cause high expression of RANKL in osteoblasts and bone stromal cells. 

Furthermore, RANKL is also expressed by endothelial cells of blood vessels and promotes the uptake of TRAP-positive progenitor cells; Osteoclast progenitors of blood vessel origin attach to RANKL on stromal cells and eventually differentiate into TRAP-positive pre-osteoclast cells. The mononuclear precursor cells then migrate through the blood vessels to the connective tissue stroma and fuse to form multinucleated at the surface of the bone, where these mature osteoclast cells, with their activity at the bone level, lead to bone resorption(18). 

In a study carried out by Hong et al.(19), it was found that fibroblasts derived from syndromic OKC had a greater potential for inducing bone resorption and could be related to high levels of COX-2, RANKL/OPG ratio, and TRAP-positive multinucleate cells which can cause bone resorption. In another study carried out by Wang et al (20) , several osteoclast markers such as RANKL, TRAP, OPG, and IL-1 were examined in 30 cases of OKC. In this study, TRAP expression and simultaneous expression of TRAP and RANKL was found in 10 and 2 OKC cases respectively.

These TRAP and RANKL-positive cells were found in the connective tissue in the vicinity of bone (between bone and capsule) and it was mentioned that these factors are involved in the activity and differentiation of pre-osteoclasts to osteoclasts and consequently bone resorption in OKC. Similarly in the present study, TRAP expression was observed in the connective tissues of the cyst wall in OKC, RC, and DC. TRAP-positive mononuclear cells were found in the perivascular region in some cases, indicating that these cells were probably osteoclast precursors with hematopoietic origin in cystic lesions. However, contrary to the mentioned studies that, the TRAP expression was reported only in the connective tissue of OKC, in our study, TRAP expression was also significantly observed in the lining epithelium of OKC.

## Conclusion

 According to the result of the present study, a higher expression of TRAP was found in the lining epithelium of OKC, which could indicate that, unlike other cysts, both the OKC epithelium and connective tissue act as a source of TRAP. This could be a reason for the higher resorption activity and more aggressive behavior of OKC compared to other cysts. 
